# Doctor, I fractured my ankle. When can I return to play? An updated systematic review

**DOI:** 10.1093/bmb/ldac016

**Published:** 2022-04-30

**Authors:** Amit Sinha, Greg Robertson, Nicola Maffulli

**Affiliations:** Department of Trauma and Orthopaedic Surgery, Ysbyty Gwynedd, Penrhosgarnedd, Bangor, Gwynedd LL57 2PW, UK; Department of Trauma and Orthopaedic Surgery, Wales Deanery, Ty Dysgu, Cefn Coed, Nantgarw CF15 7QQ, UK; Department of Orthopaedic Surgery, Queen Elizabeth University Hospital, 1345 Govan Road, Glasgow G51 4TF, UK; Department of Medicine, Surgery and Dentistry, University of Salerno, Via S. Allende, 84081 Baronissi, Salerno, Italy; School of Pharmacy and Bioengineering, Keele University School of Medicine, Thornburrow Drive, Stoke on Trent ST4 7QB, UK; Centre for Sports and Exercise Medicine, Queen Mary University of London, Barts and the London School of Medicine and Dentistry, Centre for Sports and Exercise Medicine, Mile End Hospital, 275 Bancroft Road, London E1 4DG, UK

**Keywords:** ankle fractures, trauma, return to sport, rehabilitation, conservative management, surgical management

## Abstract

**Introduction:**

Ankle fractures in sport are common. Their optimal management is unclear, as is when patients can return to their sports activities. This systematic review provides a contemporary assessment of the literature on return to sports following acute traumatic ankle fractures managed both operatively and non-operatively.

**Sources of data:**

We systematically searched Pubmed, Google Scholar, the Cochrane Library, EMBASE and CINAHL using the terms ‘ankle fractures’, ‘ankle injuries’, ‘athletes’, ‘sports’, ‘return to sport’, ‘return to activity’, ‘operative management’, ‘non-operative management’.

**Areas of agreement:**

Thirteen retrospective studies fulfilled the inclusion criteria. The methodological quality of the studies was generally poor. The proportion of patients returning to sporting activity was high. In some studies, a quicker return to sporting activity was demonstrated in patients managed non-operatively.

**Areas of controversy:**

The time to return to sporting activity and level of performance post-treatment are not universally recorded, and the optimal time to return to sport remains to be confirmed.

**Growing points:**

Conservative management for stable or undisplaced fracture may result in a higher proportion of patients returning to sport more quickly.

**Areas timely for developing research:**

Randomized controlled trials should compare conservative to surgical treatment for appropriately chosen fracture patterns. Future studies should routinely report the timing of return to sport, the level of performance reached, and the time to achieve this.

## Introduction

Ankle injuries can occur in both competitive and recreational athletes, with a reported prevalence of 15–25% of all sports injuries.^1–3^ Acute traumatic ankle fractures account for between 7% and9% of all acute sport-related fractures.^4^ Return to sport is an important goal in treatment of ankle fractures in a sporting population.^5^ Open reduction and internal fixation, as a treatment option for the management of ankle fractures,^6^ especially for athletes, may allow earlier mobilization and hence a quicker return to sports.^7^ However, non-operative management can be used successfully for ‘stable’, undisplaced ankle fractures in athletic patients.^8–9^

Only one previous systematic review, published in 2013, assessed return to sport after ankle fractures.^10^ That review only included operatively-managed ankle fractures, and assessed the outcome of both acute and stress fractures.

The present updated systematic review provides a contemporary assessment of the literature on the return to sports following acute ankle fractures. The present systematic review focuses exclusively on acute traumatic sport-related ankle fractures, and reviews the outcomes of both operative and non-operative management. The outcome measures of interest are the rate of return to sport, the time taken to return to sport and the level of sporting activity achieved post-treatment. Predictive factors such as fracture pattern, age and gender that may have an effect on the return to sporting activity will also be assessed.

## Materials and methods

The study was performed adhering to the PRISMA guidelines (preferred reporting items in systematic reviews and meta-analysis).^11^

**Fig. 1:**
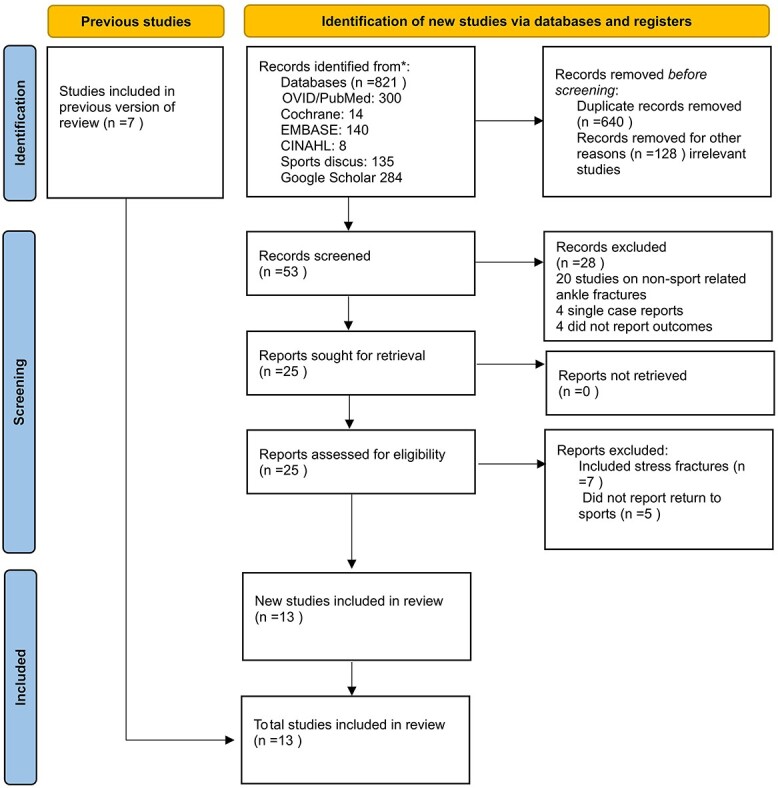
Selection of articles for inclusion in the review in accordance with the PRISMA protocol.

A search was performed in January 2022 of the online databases Pubmed, Google Scholar, the Cochrane Library, EMBASE and CINAHL and Sports Discus using a combination of the following terms: ‘ankle fractures’, ‘ankle injuries’, ‘athletes’, ‘sports’, ‘return to sport’, ‘return to activity’, ‘operative management’, ‘non-operative management’. The reference lists of all articles identified in the electronic search were then hand searched to identify further relevant literature which may have been missed in the electronic search. We excluded articles which assessed stress fractures of the ankle, cadaver studies, biomechanical studies, and single case reports. Articles published in English, Italian, French and Spanish were included based on the linguistic ability of the authors. Two authors (AS and GR) were involved in deciding which articles were included. This included independent review of every abstract, review of every full text and reference list. The search strategy is displayed in Figure 1.

From the articles included, information regarding patient demographics, fracture type and associated injuries, treatment technique, time to return to sporting activity, level of performance reached, patient reported outcome measures were extracted. Predictive factors for return to sporting activity such as fracture pattern and treatment technique were also sought. The specified treatment methods comprised either open reduction and internal fixation (ORIF) or conservative management.

The methodological quality of each included study was assessed using the modified Coleman Methodology Score (mCMS).^12^ Each study was scored for each of the ten criteria to give an overall score out of 100. A score of 100 indicates a well-designed study, which limits the effect of bias and confounders.

A summary of the study findings is displayed in Table 1 below.

**Table 1:** Study findings

Author	Number fractures	Fracture type	Treatment	% return to sport	Time to return to sport	Patient reported outcome measure
Werner *et al.*^13^	237	197 isolated distal fibula. 40 combined (distal fibula plus ankle dislocation or distal tibia fracture.)	ORIF 128 cases. (100 in isolated distal fibula group and 28 in combined group.) Conservative 109 cases.	90%	Total Cohort:—ORIF group 123.8 days—Conservative group 75.3 days. Within isolated distal fibula fracture group:—ORIF- 117.1 days—Conservative- 72.1 days Irrespective of treatment type:—Isolated distal fibula fracture 93.6 days—Combined injury 132.2 days	Unreported
Brent Lievers *et al.*^14^	49	28 isolated lateral malleolus 21isolated medial malleolus	ORIF 20:—Lateral malleolus 13—Medial malleolus 7 Conservative: 28	100%	Lateral malleolus: 65 days Medial malleolus: 38 days	Unreported
Chiet Hong *et al.*^15^	31	31 trimalleolar fractures. Subdivided into: 23 Weber B 8 Weber C	ORIF 31 cases	75%	Unreported	Olerud and Molander score. Overall mean score 78.3 at 1 year. Posterior malleolus fixed: 78.7 Posterior malleolus not fixed: 77.3 Weber B: 83.9 Weber C: 67.1
Orr *et al.*^16^	72	Isolated lateral malleolus: 45 Isolated medial malleolus: 11 Bimalleolar: 14 Trimalleolar: 2	ORIF 72 cases	64%	Unreported	Unreported
Robertson *et al.*^17^	96	Isolated lateral malleolus: 45 Isolated medial malleolus: 5 Bimalleolar: 34 Trimalleolar: 12	52 cases non-operativel Y(43 lateral malleolus, 2 medial malleolus, 7 bimalleolar). 44 operativel Y(2 lateral malleolus, 3 medial malleolus, 27 bimalleolar, 12 trimalleolar)	94% overall. 100% non-operative group 87% operative group	26 weeks Non-operative group: 20 weeks Operative group: 35 weeks Syndesmotic injury: 43 weeks Non-syndesmotic injury:25 weeks	Unreported
Chiet Hong *et al.*^18^	47	Bimalleolar: 26 Trimalleolar: 21	ORIF: 47 cases	81.8% 27.3% to pre-injury level	Unreported	Mean Olerud and Molander score 82.1. Bimalleolar: 81.7 Trimalleolar: 78.3 VAS Bimalleolar: 1.8 VAS Trimalleolar: 2
Colvin *et al.*^5^	243	Numbers in each category not specified other than 83 trimalleolar fractures	ORIF 243	25% overall 88% recreational athletes. 12% competitive athletes	Unreported	Short Musculoskeletal Functional Assessment (SMFA) and American Orthopaedic Foot and Ankle Society score (AOFAS) indicated delay in functional recovery with regards to age, female gender and presence of syndesmotic injury.
Porter *et al.*^19^	27	Bimalleolar: 10 Isolated lateral malleolus: 6 Salter-Harris Type: 4 Isolated medial malleolus: 2 Pilon: 1	ORIF 27 cases	96% One case of non-return.	Isolated lateral malleolus: 6.8 weeks. Isolated medial malleolus: 17.0 weeks	AAOS Lower Limb Core Modules 94.6 for function 98.0 for pain
Mai *et al.*^20^	42	Subtypes not reported.	ORIF: 42 cases.	78.6%	Unreported	Unreported
Walsh *et al.*^21^	4	Case 1- Distal fibula fracture with deltoid ligament injury. Case 2- Distal fibula fracture with deltoid ligament injury. Case 3- Trimalleolar ankle fracture. Case 4- Syndesmosis injury	Case 1: Rush rodding of fibula and deltoid suturing. Case 2: Suturing of deltoid plus cast immobilization. Case 3: ORIF Case 4: Syndesmosis fixation with 2 screws.	100%	Unreported	Unreported
Donley *et al.*^22^	3	High fibula fracture with syndesmosis injury	ORIF 3 cases	100%	Unreported	Unreported
Navarro Garcia *et al.*^23^	60	Weber A: 9 Weber B: 38 Weber C: 13	ORIF 60 cases	100%	Unreported	Unreported
Pina *et al.*^24^	92	Weber A: 9 Weber B: 37 Weber C: 46 Posterior malleolus involved in 24 cases.	ORIF 92 cases	91%	Unreported	Mean AOFAS 90.93.

## Results

A total of 13 studies were identified. All focused on clinical and functional outcomes for a sporting population following ankle fracture (Table 1).

The mean modified Coleman Methodology score was 44, indicating a relatively poor methodological quality of the included studies. All studies scored poorly for study type, as they were all retrospective case series. Further areas which were uniformly poor were study size and duration of follow up. Operative technique and post-operative rehabilitation were either described in significant detail or very briefly. The Coleman Methodology Score for each study is displayed in Table 2.

**Table 2:** Modified Coleman methodology score

Author	Modified Coleman methodology score
Werner *et al.*^13^	35
Brent Lievers *et al.*^14^	30
Chiet Hong *et al.*^15^	42
Orr *et al.*^16^	37
Robertson *et al.*^17^	50
Chiet Hong *et al.*^18^	54
Colvin *et al.*^5^	63
Porter *et al.*^19^	50
Mai *et al.*^20^	45
Walsh *et al.*^21^	36
Donley *et al.*^22^	37
Navarro Garcia *et al.*^23^	34
Pina *et al.*^24^	60

### Injury pattern

The included studies assessed a variety of fracture pattern, and some assessed the impact of associated syndesmotic injury. Fractures were confirmed radiographically in all studies.

Werner *et al.*^13^ assessed the incidence and outcome of distal fibular fractures reported to the National Football League (NFL) Injury Surveillance System over a 14-year period from 2000 to 2014. Isolated distal fibula fractures as well as those combined with dislocation of the ankle joint and or a distal tibial fracture were included. Syndesmotic injuries were not included. One hundred and ninety-seven cases of isolated distal fibula fracture were described along with 40 combined injuries. One hundred and twenty-eight fractures were managed surgically.

Brent Lievers *et al.*,^14^ in a retrospective epidemiological study, identified the most common foot and ankle injuries among collegiate American football players by identifying data from the NCAA (National Collegiate Athletic Association) Injury Surveillance System, during two seasons of play. Regarding ankle fractures, injuries were described as either medial malleolus fractures or lateral malleolus fractures. 28 lateral malleolus fractures were identified along with 21 medial malleolus fractures. Twenty cases were managed surgically.

Chiet Hong *et al.*^15^ retrospectively assessed the outcome following surgical management of 31 trimalleolar ankle fractures. With regards to the fibula component of this injury, the Weber classification system was used, with 23 Weber B and 8 Weber C fractures. No syndesmosis injury was described.

Orr *et al.*^16^ assessed the return to running in a military cohort, following ORIF for ankle fracture. Forty-five lateral malleolus, 11 medial malleolus, 14 bimalleolar and 2 trimalleolar fractures were identified. Thirty-three patients had suffered a syndesmotic injury.

Robertson *et al.*^17^ retrospectively assessed 96 sports-related ankle fractures which presented over one year. These included 45 lateral malleolus fractures, 5 medial malleolus fractures, 34 bimalleolar fractures, 12 trimalleolar fractures. Five syndesmotic injuries were identified. Fifty-two fractures were managed surgically, and 44 conservatively.

Chiet Hong *et al.*^18^ compared the outcome between operatively-managed bimalleolar and trimalleolar fractures. Twenty-six bimalleolar fractures and 21 trimalleolar fractures were included. No patients with a syndesmosis injury were included.

Colvin *et al.*^5^ assessed the outcomes of 488 operatively treated ankle fractures, of which 243 were identified as occurring in patients participating in sporting activity prior to injury. Eighty-three trimalleolar fractures were described along with 54 cases of a syndesmosis injury. No further classification of the fractures was given.

Porter *et al.*^19^ reviewed 27 cases of sport-related ankle fracture in young athletes, all managed operatively. This study had the widest range of fracture patterns, with 10 bimalleolar fractures, 6 isolated lateral malleolus fractures, 4 isolated medial malleolus fractures, 4 Salter-Harris type fractures, 1 pilon fracture and 4 syndesmotic injuries. The mean age of the patients was 18.1 years with a range of 5.9 years. Thus there were some skeletally immature patients, though only 4 physeal injuries were described.

Mai *et al.*^20^ described 42 cases of operatively-managed ankle fractures, though the injury subtypes were not described.

Walsh *et al.*^21^ reported on an operative case series of two distal fibula fractures with deltoid ligament injury, one trimalleolar fracture and one syndesmosis injury.

Donley *et al.*^22^ presented three cases of high fibula fractures with syndesmosis injury, all operatively managed. The authors described the injuries using the Lauge–Hansen^25^ classification system.

Navarro Garcia *et al.*^23^ and Pina *et al.*^24^ both described fractures according to the Danis–Weber classification system.^25^ All fractures were operatively managed in both studies.

### Return to sporting activity

The return to sporting activity was assessed as the proportion of patients in each study who returned to sporting activity. The time taken to return to sporting activity and the level of performance achieved post-fracture were not universally reported. There appears to be a range of return to sporting activity from 25%^5^ to 100%.^14^^,^^21^^,^^22^^,^^23^ Colvin *et al.*^5^ only reported a 25% return rate with a much higher rate of return among recreational athletes, at 88%.

Werner *et al.*^13^ and Robertson *et al.*^17^ assessed the outcomes following both conservative and surgical treatment. Both studies demonstrated a high rate of return to sporting activity with 90% and 94% overall return rates, respectively. Only Robertson *et al.*^17^ compared return to sporting activity between the conservative and surgical group: they found a higher return rate to sports in the conservatively managed group, with 100% compared with 87%. Werner *et al.*^13^ did not compare the return rate to sport between the surgical and conservative group. However, the surgical group had a higher number of days lost to sporting activity (123.8 days) compared to the conservative group (75.3 days).

The reported times taken to return to sporting activity ranged from 7 to 43 weeks. The quickest time was reported by Brent Lievers *et al.*^14^ for the isolated medial malleolus fracture group. The time to return to sport was not compared between operated and conservatively managed cases within this group. The slowest time to return to sport was reported by Robertson *et al.*,^17^ with 43 weeks for operatively managed ankle fractures with associated syndesmotic injury. Werner *et al.*^13^ and Robertson *et al.*^17^ both reported a statistically significant quicker return to sporting activity in conservatively managed compared to surgically managed fractures.

Very few studies described outcome based on injury pattern. Of those that did, Werner *et al.*^13^ described a quicker return with an isolated distal fibula fracture compared with a distal fibula fracture combined with ankle dislocation or distal tibia fracture. Brent Lievers *et al.*^14^ and Porter *et al.*^19^ described results for isolated medial and isolated lateral malleolus fractures. Interestingly they found different patterns in return to sport. Brent Lievers at al^14^ reported a quicker return with an isolated medial malleolus fracture whereas Porter *et al.*^19^ reported a quicker return with an isolated lateral malleolus fracture. Robertson *et al.*^17^ described a quicker return to sport in patients without an associated syndesmotic injury.

The level of performance following return to sporting activity was not reported in all studies. Chiet Hong *et al.*^18^reported that only 27.3% of patients returned to pre-injury level of performance. Pina *et al.*^24^ reported that 21.8% of their patients returned but with limitations. Mai *et al.*^20^ assessed the functional outcomes of NFL athletes who had a range of orthopaedic injuries and operations, and reported that patients with an ankle fracture experienced a reduction in performance level at one year post injury, but that this returned to baseline at 2–3 years. This might suggest that patients who have higher functional demands at baseline may take longer to return to pre-injury function if at all. The findings from Colvin *et al.*^5^ would also support this finding in that only 12% of competitive athletes returned to sport compared with 88% recreational athletes.

### Patient reported outcome measures

Some of the studies used patient reported outcome measures to assess outcome. Chiet Hong^15–18^used the Olerud and Molander scale^26^ and Visual Analogue Scale (VAS).^27^ The American Orthopaedic Foot and Ankle Society Score (AOFAS)^28^was used by Colvin *et al.*^5^ and Pina *et al.*.^24^ Colvin et^5^ used the Short Musculoskeletal Functional Assessment (SMFA)^29^whereas Porter *et al.*^5^ used the American Academy of Orthopaedic Surgeons (AAOS) Foot and Ankle Core Modules.^30^

## Discussion

The key findings from the present updated systematic review were that most studies reported a high rate of return to sporting activity following an acute traumatic ankle fracture. However, the rate of return to pre-injury level of performance was not as positive, particularly for the higher demand patients. Two studies also reported findings which suggest that conservative management, for ‘stable’ undisplaced fracture, may result in a higher proportion of patients returning to sporting activity, more quickly.

Several studies reported a high rate of return to sporting activity following acute ankle fractures, with 4 of 13 reporting a 100% return rate,^14^^,^^21^^,^^22^^,^^23^ and 5 of 13 reporting a return rate of over 80%.^13^^,^^17^^,^^18^^,^^19^^,^^24^ The present updated systematic review also included studies assessing the role of conservative management. One study highlighted that a higher proportion of patients managed conservatively returned to sporting activity, as compared to patients managed surgically.^17^ Surgical management is indicated in displaced, unstable ankle fractures: this suggests that operative treatment may have longer term morbidity regarding return to activity, compared to conservative management. Furthermore, two studies highlighted a quicker return to sporting activity following conservative treatment.^13^^,^^17^ Such findings suggest that conservative treatment may be encouraged for ‘stable’, undisplaced ankle fractures in athletic patients. However, these studies were retrospective and descriptive epidemiological studies. The patients in each treatment modality were not matched at baseline for demographics or fracture pattern. Thus, the effect of confounding factors was not accounted for; hence, the differences between the conservative and the surgical groups could result from several factors, including chance.

The present updated systematic review aimed to ascertain whether there are any predictive factors for return to sports following an acute ankle fracture. There appears to be some conflicting evidence for this. Colvin *et al.*^5^ demonstrated that older age, female gender and additional syndesmotic injury were predictive factors for a delayed return to sporting activity. Robertson *et al.*^17^ demonstrated a delayed return to sports following a fracture associated to a syndesmosis injury. However, some of the other studies did not show this association.^16^^,^^19^ Pina *et al.*^24^ suggested that the presence of an osteochondral lesion in addition to fracture, rather than a syndesmosis injury, results in a greater chance of adverse outcome on return to sports. Furthermore, there was heterogeneity in the fracture patterns described, and few studies assessed outcome based on fracture configuration. Therefore, it is difficult to accurately ascertain whether there are strong predictive factors with regard to patient demographics and fracture configuration for a delayed return to sports following an acute ankle fracture.

The difficulty of ascertaining predictive factors is one of the limitations of the present updated systematic review. A further limitation is that it is difficult to draw conclusions on the timing of return to sports activities following these injuries. There is variability in the way this outcome is reported among the various studies, with some studies not even specifying the time to return to sport. Similarly, some studies did not specify the level of performance reached post-treatment. In such instances, it can be difficult to determine the effectiveness of the treatment given.

The methodological quality of the studies assessed in the present systematic review is poor: all included studies were retrospective. There are no randomized controlled trials in the management of acute sport-related ankle fractures. This could be an area of future research, with consideration given to comparing conservative to surgical management in appropriate fracture types.

A further area to be developed is sport-specific return following ankle fractures. Most of the studies did not specify the nature of the sporting activity undertaken. There were a few studies specifically focused on American Footballers, with information drawn from injury surveillance databases. It would be very beneficial if other sports developed similar databases, allowing such information to be obtained on recovery and return to play in different sports. Such sport-specific information would be useful, both for clinicians and athletes, and could help manage the athlete’s expectations on return to sport.

There is a definite proportion of athletes who, despite what appears to be optimal treatment of their ankle fractures, did not return to sport, or returned to sport at a lower level than expected. Further observation and assessment of these patients is required to ascertain what may have caused such an outcome. This may help to identify sub-optimal treatment strategies, and guide clinicians to avoid them.

Ideas for further research in this area include the need for randomized controlled trials to compare conservative to surgical treatment options, for appropriately chosen fracture patterns. Consistency in reporting in any future investigation is also required, with regards to timing of return to sport: the level of performance reached and the time to achieve this should be consistently specified. Prospective studies which stratify for demographics such as age, gender and associated soft tissue injury may help to identify predictive factors for the outcome of these injuries.

Sports-specific outcome research is also an area for future development. Some of the studies included in the present systematic review have provided limited data on this, with some sport-specific databases active at present. However, a more widespread application of such databases may help to identify sports-specific treatment options. In addition to this, it would be worthwhile to conduct further research into those athletes who failed to return to sport, or returned at a lower level than expected, to identify potentially sub-optimal treatment choices.

## Conclusion

Most studies report high rate of return to sports following acute ankle fractures. Conservative management, classically indicated for ‘stable’ undisplaced fractures, may result in a higher proportion of patients returning to sporting activity, and returning more quickly though it must be noted that conservatively managed fractures are inherently more stable and have less injury.

## Data availability

Data will be made available upon reasonable request to the Corresponding Author.
